# Gamma-gliadin specific celiac disease antibodies recognize p31-43 and p57-68 alpha gliadin peptides in deamidation related manner as a result of cross-reaction

**DOI:** 10.1007/s00726-021-03006-7

**Published:** 2021-05-31

**Authors:** Ádám Diós, Rita Elek, Ildikó Szabó, Szilvia Horváth, Judit Gyimesi, Róbert Király, Katharina Werkstetter, Sibylle Koletzko, László Fésüs, Ilma R. Korponay-Szabó

**Affiliations:** 1 Department of Pediatrics, Faculty of Medicine, University of Debrecen, Debrecen, Hungary; 2 Doctoral School of Molecular Cell and Immune Biology, University of Debrecen, Debrecen, Hungary; 3 Coeliac Disease Center, Heim Pál National Pediatric Institute, Budapest, Hungary; 4 Department of Biochemistry and Molecular Biology, Faculty of Medicine, University of Debrecen, Debrecen, Hungary; 5 Division of Gastroenterology and Hepatology, Department of Pediatrics, Dr. Von Hauner Children’s Hospital, University Hospital, Ludwig-Maximilian’s University Munich, Munich, Germany; 6 Department of Pediatrics, Gastroenterology and Nutrition, School of Medicine Collegium Medicum, University of Warmia and Mazury, Olsztyn, Poland

**Keywords:** Celiac disease, Gliadin, Deamidation, Antibody, Serum reactivity, Cross-reactivity

## Abstract

Celiac disease (CeD) is a T-cell-dependent enteropathy with autoimmune features where tissue transglutaminase (TG2)-mediated posttranslational modification of gliadin peptides has a decisive role in the pathomechanism. The humoral immune response is reported to target mainly TG2-deamidated γ-gliadin peptides. However, α-gliadin peptides, like p57-68, playing a crucial role in the T-cell response, and p31-43, a major trigger of innate responses, also contain B-cell gliadin epitopes and γ-gliadin like motifs. We aimed to identify if there are anti-gliadin-specific antibodies in CeD patients targeting the p31-43 and p57-68 peptides and to examine whether deamidation of these peptides could increase their antigenicity. We explored TG2-mediated deamidation of the p31-43 and p57-68 peptides, and investigated serum antibody reactivity toward the native and deamidated α and γ-gliadin peptides in children with confirmed CeD and in prospectively followed infants at increased risk for developing CeD. We affinity-purified antibody populations utilizing different single peptide gliadin antigens and tested their binding preferences for cross-reactivity in real-time interaction assays based on bio-layer interferometry. Our results demonstrate that there is serum reactivity toward p31-43 and p57-68 peptides, which is due to cross-reactive γ-gliadin specific antibodies. These γ-gliadin specific antibodies represent the first appearing antibody population in infancy and they dominate the serum reactivity of CeD patients even later on and without preference for deamidation. However, for the homologous epitope sequences in α-gliadins shorter than the core QPEQPFP heptapeptide, deamidation facilitates antibody recognition. These findings reveal the presence of cross-reactive antibodies in CeD patients recognizing the disease-relevant α-gliadins.

**Supplementary Information:**

The online version contains supplementary material available at 10.1007/s00726-021-03006-7.

## Introduction

Celiac disease (CeD) is a chronic, T-cell mediated enteropathy with autoimmune features induced by gluten ingestion in genetically susceptible people. Gluten is the external dietary antigen that triggers the coupled production of anti-gliadin peptide-specific antibodies and anti-tissue transglutaminase autoantibodies (anti-TG2) through disease progression (Sollid and Jabri 2013).

Gluten comprises the prolamin proteins found in certain cereals: wheat (gliadin, glutenin), barley (hordein) and rye (secalin) (Balakireva and Zamyatnin 2016). Among cereals, wheat gliadin molecules contain the most immunogenic sequence motifs. Gliadin molecules can be divided into three subcategories: α/β, γ and ω-gliadins. Gliadins as prolamin proteins contain high amounts of proline and glutamine residues in the form of repeated sequence motifs, and this renders them resistant to the human intestinal proteases and preserves their antigenic nature (Shan et al. 2005).

CeD is a typical example where posttranslational modification plays a key role in the pathogenesis of human diseases. The immunogenicity of non-degradable gliadin peptides is enhanced by human tissue transglutaminase (TG2)-mediated deamidation, preferentially occuring at QXP, QXPF/Y or QXXF/Y sequence motifs (Vader et al. 2002). Selective deamidation introduces a negative charge which increases the binding of gliadin peptides to HLA DQ2, DQ8 molecules, thereby facilitates their presentation to T lymphocytes and the activation of the adaptive anti-giadin response (Qiao et al. 2005). The presence of DQ2 and/or DQ8 is indispensable, however, not sufficient for CeD to develop (additional non-HLA susceptible genes and environmental factors are required). The best-studied immunodominant DQ2-restricted T-cell epitope is the p57-68 α-gliadin fragment (Arentz-Hansen et al. 2000), also forming part of the 33-mer most potent T-cell activating sequence (Qiao et al. 2004).

Besides T-cell activation, gliadin peptides also evoke humoral immune responses. The B-cell recognition is not HLA dependent and usually presupposes conformational epitopes, however, in the case of gliadin peptides, the identified B-cell epitopes are linear and overlap with the T-cell stimulatory sequences (Dørum et al. 2016). Based on peptide library screening studies, the PEQ and PFP are the most common B-cell stimulatory motifs (Choung et al. 2016), and in line with that, the QPEQPFP γ-gliadin derived sequence is a highly disease-specific immunodominant B-cell epitope (Osman et al. 2000; Schwertz et al. 2004).

The α-gliadin fragment p31-43 has a unique toxic effect in in vitro experiments, causing higher proliferation rate, actin rearrangement, reactive oxygen species production, TG2 activation and interleukin-15 production in Caco2 cell line and CeD patients-derived biopsy specimens (Barone et al. 2014). This peptide fragment is thought to escape T-cell presentation since it is retained in the early endosomal compartment and cannot enter the lysosomal pathway (Barone et al. 2010).

In the literature 8 α and 13 γ-gliadin derived CeD relevant T-cell epitopes have been identified (Sollid et al. 2020) and they are equally potent immunogens based on T-cell stimulatory experiments (Qiao et al. 2005; Tollefsen et al. 2006; Dørum et al. 2010). However, B-cells preferentially recognize the γ-gliadin sequences compared to α-gliadins (Srinivasan et al. 2013; Dørum et al. 2016).

According to previous findings, α-gliadin peptides p31-43 and p57-68 both contain a potential B-cell epitope (QXQPFP) (Osman et al 2000*)*. Since the major fraction of T-cells recognize p57-68, a selection force by T-cell help can be considered for B-cells of similar specificity.

In our study, we aimed to identify whether antibodies in CeD patients specifically recognize p31-43 toxic and p57-68 T-cell stimulatory peptides or their TG2 deamidated forms. Taking advantage of patient cohorts previously well characterized by extensive serologic assays and T-cell studies, we show here in real-time direct peptide binding assays and by means of single peptide-purified antibodies that the main γ-gliadin specific celiac antibody population may cross-react with the p31-43 and p57-68 peptides, especially with their deamidated forms.

## Materials and methods

### Patients

Serum samples from 69 Hungarian pediatric patients with untreated CeD (median age 6.2 years, range 1.8–17.3) who had participated in the ProCeDE study evaluating diagnostic accuracy in anti-TG2 positive subjects (Werkstetter et al. 2017) and from 21 patients who had participated in the PreventCD prospective follow-up study of genetically predisposed newborns having a first-degree family member with CeD (http://www.preventceliacdisease.com, Vriezinga et al. 2014) were used. CeD was diagnosed with a small intestinal biopsy showing Marsh III lesion. Enrolment and sample collection were performed upon informed consent from the parents that their child’s serum will be used for different antibody testings related to CeD in different laboratories. Ethical permissions to the original studies were given by the committees of the internationally coordinating institutions and also locally by the Ethical Committee of the Heim Pál National Pediatric Institute, Budapest, Hungary, which also gave permission for the reuse of already collected serum samples in the present study.

### Peptides and reagents

Synthetic gliadin peptides with N-terminal biotinylation and > 95% purity were obtained from GenScript (Leiden, Netherlands) with the following sequences: γ-Glia_Q (SGGPLQPQQPFP), γ-Glia_E (SGGPLQPEQPFP), γ-Glia_sh (SGGPEQPFP), p31-43 (LGQQQPFPPQQPY), p31-43_E34 (LGQEQPFPPQQPY), p31-43_E40 (LGQQQPFPPEQPY), p57-68 (QLQPFPQPQLPY), p57-68_E65 (QLQPFPQPELPY) and an irrelevant peptide sequence (VVKVGGSSSLGW) as the negative control. All other reagents were purchased from Sigma (St. Louis, MO, USA) except if indicated otherwise.

### Analysis of gliadin peptide deamidation by liquid chromatography–mass spectrometry (LC–MS/MS)

Deamidation reaction of synthetic gliadin peptides p31-43 and p57-68 was performed in MOPS buffer (50 mM, pH 6.8) supplemented with 5 mM CaCl_2_ and 1 mM dithiothreitol applying 50 pmol of human recombinant TG2 (natural ^224^Val form) enzyme (Kanchan et al. 2013). The reaction was performed at 37 °C for 120 min at a TG2:gliadin peptide molar ratio of 1:150. Then the reaction was stopped by heat inactivation of TG2 followed by centrifugation on Amicon ultra 10 K membrane (Merck, Darmstadt, Germany) to separate the enzyme and the gliadin peptides. Prior to LC–MS/MS analysis, gliadin peptides were further cleaned with C18 PierceTip (Thermo Scientific) according to the manufacturer’s instructions and the cleaned samples were injected onto Orbitrap Fusion tribrid mass spectrometer (Thermo Scientific) functioning in high-resolution positive ion mode. MS/MS spectra were recorded and the peptide identification was done with MaxQuant using the Andromeda search engine. N-terminal biotin was set as fixed modification and deamidation (N, Q) as variable modification. The identification of deamidation sites was assisted by annotating the *b* and *y*-fragments. Data analysis was performed with Scaffold software (Proteome Software Inc.) and additional manual curation.

### Affinity-purification of gliadin peptide-specific antibodies from CeD patient serum samples

500 µg of biotinylated synthetic gliadin peptides were immobilized to 1 mL settled Pierce™ High Capacity Neutravidin Agarose (Thermo Scientific, Rockford, IL, USA) according to the manufacturer's instructions. CeD patient’s serum was diluted two times in phosphate-buffered saline pH 7.4 (PBS) + 0.1% Tween 20 and incubated with the peptide-bound agarose for 1 h at room temperature. The antibodies were eluted with five column volumes of 100 mM glycine pH 2.5 followed by buffer change to PBS with 50 K Amicon® Ultra Centrifugal Filters (Merck, Darmstadt, Germany). Concentration was determined by Bradford assay (Biorad Laboratories, Hercules, CA, USA) using human IgG as a standard.

### Serum anti-gliadin peptide antibody quantification and determination of the relative binding rates of the affinity-purified antibodies by the BLItz system

In addition to already available clinical enzyme-immunoassay results in the ProCeDE and PreventCD study, bio-layer interferometry (Personal Assay BLItz System, PALL FortéBio, Fremont, CA, USA) was used to quantitate binding of serum antibodies and of purified antibodies to single peptides. Bio-layer interferometry (BLI) is a real-time label-free optical measurement tool detecting the shift of the wavelength (nm/s) of reflected light upon binding of interactants to the sensor surface and particularly suited to measure interaction with small molecules. 3 µM of biotinylated peptides were immobilized to Streptavidin (SA)-coated biosensors (FortéBio, Fremont, CA, USA) in PBS + 0.1% Tween 20 (PBST) used as running buffer. After taking a baseline with a sensor dipped in PBST, signals were registered with a series of known concentrations in PBST of antibodies affinity-purified by the given single peptides (as described above) to construct a calibration curve for each target peptide antigen. Serum samples diluted forty times in PBST were measured in duplicates in 5 min binding assays and signals were processed with the BLItz Pro 1.2.1.5. software which calculated the reactive antibody concentration (µg/mL) from the corresponding calibration curve. Biosensors were regenerated in 0.5 M phosphoric acid and reused up to ten times. Previous experiments showed no decrease of binding to sensors regenerated up to 20 times in this way. Control experiments with and without serum samples and then applying anti-human immunoglobulin antibodies (anti-IgG or IgA, Dako, Roskilde, Denmark) as secondary antibodies indicated that the label-free BLI measurement determines antibody binding.

To test the potential cross-reactivity of antibodies affinity-purified with a given gliadin peptide antigen, we determined their relative bindings to each gliadin peptide at equal antibody concentrations. The measurements were performed in 5 min assays in duplicates on SA biosensors coated with 3 µM of gliadin peptides using antibodies diluted to 150 nM in PBST. The obtained signal (nm/s) was expressed as binding rate by the BLItz Pro 1.2.1.5. software and was proportional to the amount of bound antibodies. After subtraction of binding rates to the irrelevant control peptide, duplicates were averaged and values were normalized as percentages of the binding to the peptide wherewith the given antibody reagent had been purified.

### Cell culture studies

The toxic effect of gliadin peptides was investigated as previously described (Stenman et al. 2010). Briefly, Caco-2 cells (American Type Culture Collection, Rockville, MD, USA) were grown to confluency in Nunc^TM^ 4-well dishes (Thermo Scientific) in a Minimum essential medium supplemented with 20% Fetal bovine serum (FBS), 50 U/mL Penicillin–streptomycin, 1 mM sodium pyruvate, 1.5 g/L sodium bicarbonate and 0.1 mM Non-essential amino acids. Before the experiments, FBS content was decreased to 1% for overnight and then cells were incubated with 100 μg/mL of biotinylated synthetic gliadin peptides for 24 h in 5% CO_2_ incubator at 37 °C. After 3 × washings with PBS, cells were fixed with 4% paraformaldehyde, permeabilized with 0.05% TritonX, and stained with mouse monoclonal anti-ZO-1 antibodies (1:200; Zymed Inc. San Francisco, CA, USA) for overnight at + 4 °C. AlexaFluor 488 goat anti-mouse immunoglobulin G (1:1000; Invitrogen, Carlsbad, CA, USA) were used as secondary antibodies for 90 min using extensive washings between incubation steps. Stained tight junctions were visualized with an Olympus IX81 microscope (Olympus, Hamburg, Germany).

### Statistical analyses

Differences in antibody concentrations reactive with different gliadin peptides as multiple groups were compared with *one-way* ANOVA tests. In the evaluation of the binding to native and deamidated counterparts of the same peptides, two-tailed *T*-test was used to compare the two groups and the Pearson correlation test was applied to investigate the correlation of numeric values obtained in BLItz. The Spearman's Rank correlation test was used to compare the values obtained in BLItz and clinical ELISA/ELIA measurements. A *p* value < 0.05 was considered significant. Figures were prepared using GraphPad Prism7 (San Diego, CA, USA).

## Results

### Deamidation pattern of p31-43 and p57-68 α-gliadin peptides

**Fig. 1:**
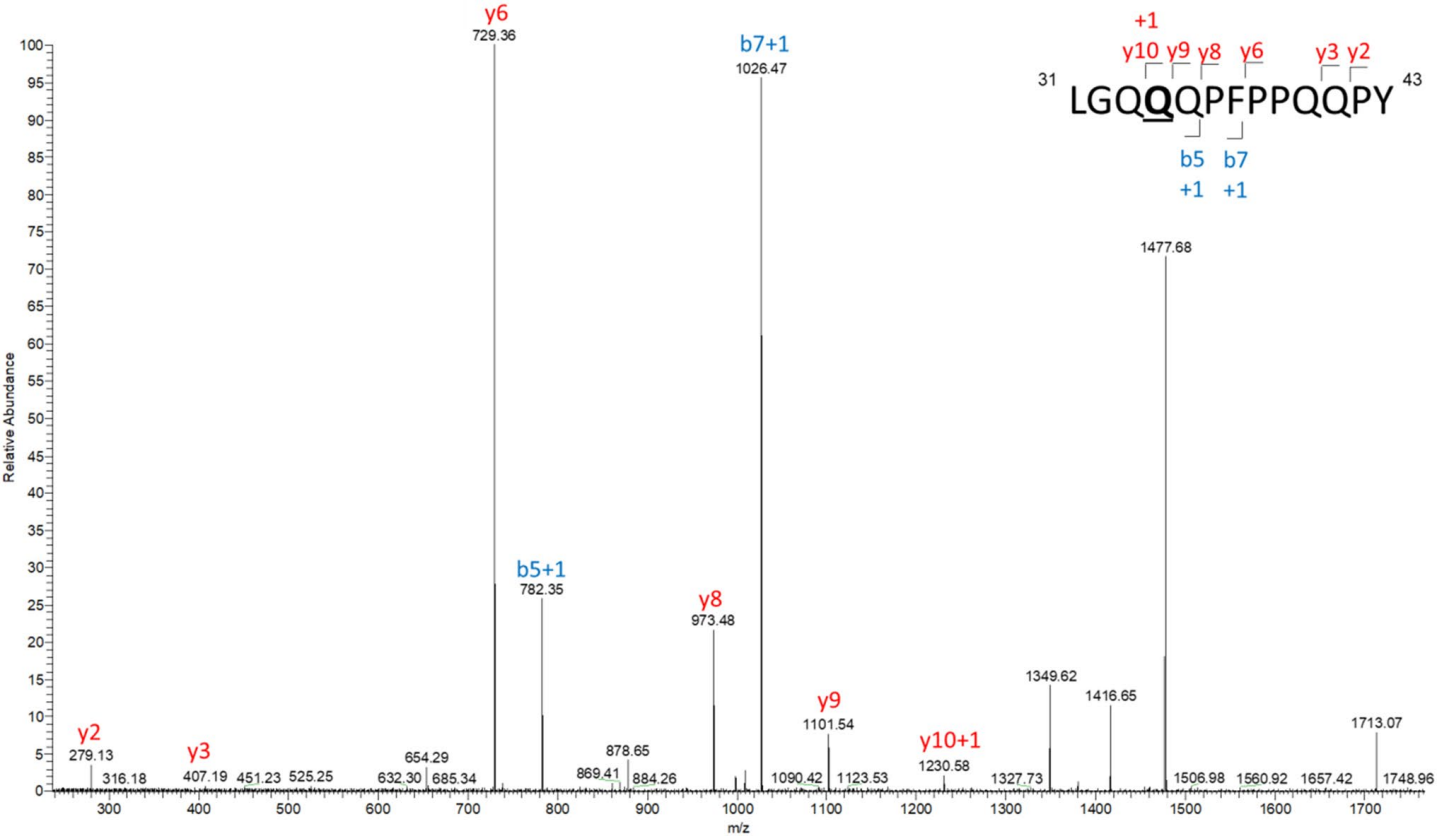
LC–MS/MS analysis of human recombinant TG2-mediated deamidation of the p31-43 peptide. 50 pmol of human recombinant TG2 enzyme were incubated with 150 fold molar excess of the p31-43 peptides for 2 h at 37 °C. The resulting deamidation products were identified by LC–MS/MS analysis, where the detected b- and y- fragments indicate a single deamidation at residue Q34 (+ 1 in the respective fragments)

**Fig. 2:**
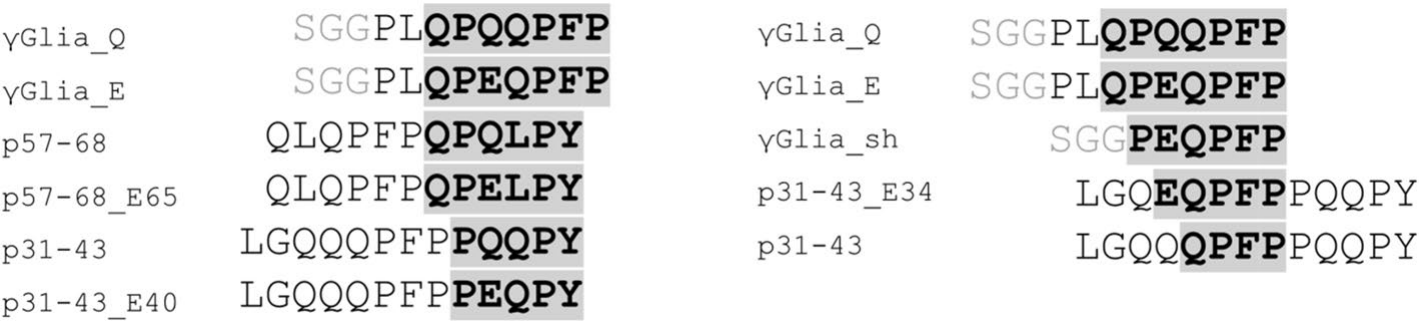
List of synthetic gliadin peptides used in our experiments. All peptides were synthesized with an N-terminal biotin tag, SGG was used as a linker sequence to ensure the comparable length of the peptides. The immunogenic γ-gliadin QPQQPFP epitope and its deamidated or shorter homologous sequences within the used peptides are highlighted with grey boxes. Peptides p31-43_E34, p31-43_E40 and p57-68_E65 bear TG2-specific deamidations (Q to E) as seen in MS/MS spectra

To directly examine the deamidation patterns of synthetic α-gliadin peptides, p31-43 (LGQQQPFPPQQPY) and p57-68 (QLQPFPQPQLPY) peptides were treated with recombinant human TG2, respectively. The resulted deamidation sites were identified by liquid chromatography–mass spectrometry (LC–MS/MS). Spectras confirmed that deamidation occurs at both single and multiple glutamine residues (Fig. 1, S1), including Q34 or Q40 in p31-43, and Q65 in p57-68, which creates relevant B-cell epitopes. Based on these results we selected the following native peptides and their deamidated counterparts (Fig. 2) for the further experiments: γGlia_Q consisting of the QPQQPFP B-cell epitope, p31-43 and p57-68 α-gliadin peptides containing the QXQPFP B-cell epitope plus PQQPF/Y mimic sequence motifs. Furthermore, we included a shortened version of the deamidated γ-Glia epitope to test whether it is also suitable for antibody recognition.

### Celiac patients’ serum antibodies target dominantly γ-gliadins, also show remarkable binding to p31-43, but poorly recognize p57-68 α-gliadin peptides

**Fig. 3:**
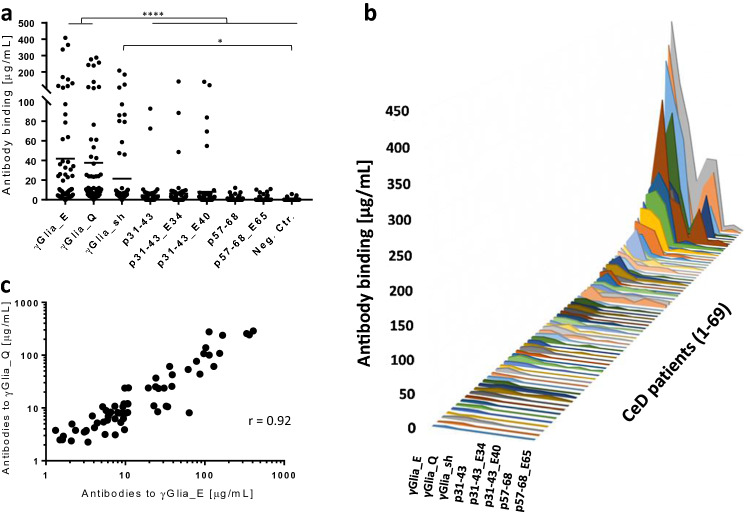
Binding of serum antibodies of untreated celiac disease (CeD) patients (*n* = 69) to biotinylated native and deamidated α- and γ-gliadin peptides. Assays were performed by bio-layer interferometry on peptide-coated Streptavidin sensors in duplicates. Average values calculated in μg/mL from peptide-specific calibration curves were plotted. Dashes indicate mean values. 2D plot (**a**) and patient by patient 3D plot (**b**). *p* value < 0.05 was taken as significant. *p < 0.05 and ****p < 0.0001 by one-way Anova analysis. **c** Correlation of individual serum antibody concentration values obtained with γGlia_E and γGlia_Q antigens, *r* value was determined by the Pearson correlation test. Neg.Ctr: irrelevant peptide

To assess the antibody binding to the native and deamidated α and γ-gliadin peptides we measured reactive antibodies from the serum of 69 patients with untreated CeD who participated in the prospective ProCeDE study and already had multiple antibody results with a large series of clinical anti-gliadin, transglutaminase and endomysial antibody assays for comparison. We utilized label-free real-time molecular interaction detection studies on streptavidin biosensors in bio-layer interferometry (BLItz) to get results in μg/mL of bound antibodies to individual synthetic biotinylated peptides. Calculations of μg/mL values were made from respective calibration curves obtained with affinity-purified anti-peptide antibodies. The BLItz method has a great advantage compared to classical ELISA tests that biosensors allow better accessibility to small peptide antigens, furthermore, BLItz is able to detect both IgA and IgG antibodies in a single step for increased sensitivity. In accordance with previous clinical results, CeD patients’ gliadin peptide reactive antibodies preferentially recognized the γ-gliadin sequences, both native and deamidated (Fig. 3a). The p31-43 α-gliadin peptides also were targets of remarkable serum reactivity, while p57-68 α-gliadin peptides exerted low, but still detectable antibody binding (*p* < 0.05 compared to irrelevant control peptide).

If we interpret serum antibody binding patterns by individual patients, no patient had higher α- than γ-gliadin reactivity. High α-gliadin reactivity occurred only in patients with even higher γ-gliadin values, while the majority of CeD patients had low anti-gliadin peptide reactivities despite strong anti-TG2/endomysial antibody positivity (Fig. 3b). Interestingly, deamidation did not increase antibody binding significantly in the case of γ-gliadins (*p* = 0.74), and binding values obtained with γGlia_Q and γGlia_E were almost identical (Fig. 3c). However, we detected a slight binding preference of CeD patients’ gliadin peptide-specific antibodies toward the deamidated forms in α-gliadins.

Serum antibody binding values to the γGlia_E peptide measured by BLItz were in very good correlation with the total anti-deamidated gliadin antibody (DGP) results obtained with 6 different commercial DGP antibody kits in clinical diagnostic assays (Fig S2). However, the α-gliadin reactivity underestimated the total antibody population (data not shown). These results together indicate that γ-gliadin specific antibodies are responsible for the majority of the total anti-gliadin serum reactivity measured in clinical assays.

**Fig. 4:**
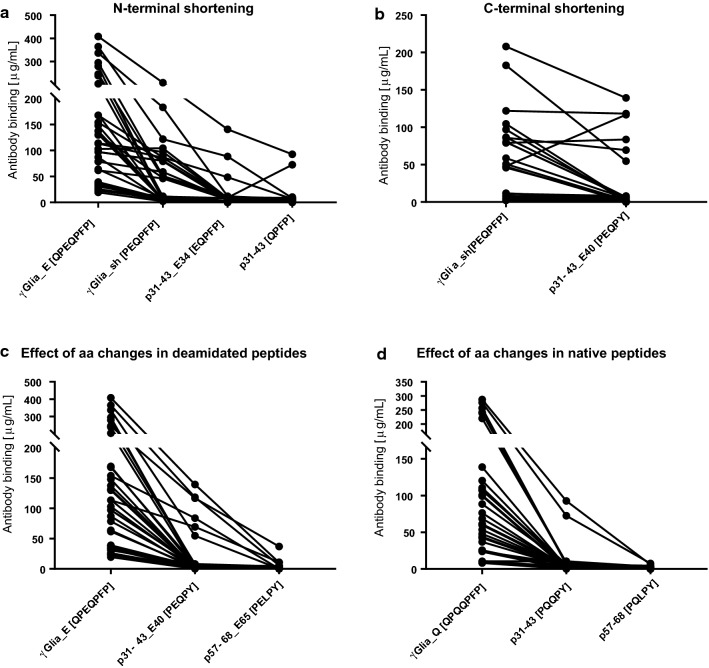
Comparison of the gliadin epitopes and their amino acid differences in relation to the measured antibody binding. Serum antibody binding results obtained by bio-layer interferometry with synthetic gliadin peptides in untreated celiac patients (ProCeDE study participants) were evaluated based on the epitope motifs found in α and γ-gliadin peptides. Only ProCeDE patients with antibody binding above 20 µg/mL for the γ-Glia_E are shown (*n* = 36). **a** Gradual decrease in the antibody binding was detected when the core heptapeptide (QPEQPFP) epitope and its homologous sequences (shown in brackets) are shorter N-terminally, or **b** when C-terminal proline is omitted and phenylalanine (F) is changed to tyrosine (Y). Effect of F to Y or glutamine (Q) to leucine (L) amino acid changes in the deamidated sequences (**c**) was less pronounced compared to the native sequences (**d**). For the complete sequences of peptides see Fig. 2. Dots denote individual patients. aa: amino acids

Regarding the evaluation of antibody binding according to the overlapping epitope motifs we found that compared to the core deamidated heptapeptide epitope QPEQPFP present in γGlia_E, peptides containing shorter homologous sequences displayed gradually reduced bindings (Fig. 4a). The N-terminally truncated PEQPFP (in γ-Glia_sh), EQPFP (in p31-43_E34), QPFP (in p31-43) sequences were associated 33.1%, 8.9%, 6.2% of antibody binding compared to the QPEQPFP (in γGlia_E) sequence, respectively. The C-terminally shorter homologous sequence PEQPY (in p31-43_E40) was associated with 43.7% of antibody binding values compared to PEQPFP (in γ-Glia_sh) (Fig. 4b).

Amino acid differences in α-gliadin peptides compared to γ-gliadins (Phe to Tyr and Gln to Leu exchange) negatively affected antibody recognition. Interestingly, these unfavourable changes were better tolerated in the deamidated set of peptides (Fig. 4c) than in the native peptides (Fig. 4d) while changing the PEQPY/PQQPY epitope (in p31-43 peptides) to PELPY/PQLPY (in p57-68 peptides) had a profound negative effect.

### Early anti-gliadin peptide antibodies target γ-gliadin peptides

To assess the early anti-gliadin peptide immune response and its evolution over time, we took advantage of the availability of blood samples from the prospective PreventCD study which followed from birth infants with high familial CeD risk. The serum samples collected at the age of 4 months (before any gluten intake), 9 months (after controlled dietary gluten intake) and 2 years (or at the time when patients started to show endomysial antibody positivity) were tested by bio-layer interferometry utilizing the same synthetic peptides in 21 children who developed CeD.

**Fig. 5:**
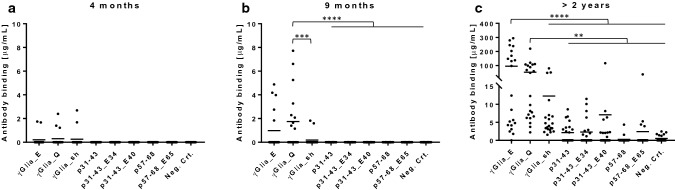
Development of serum anti-gliadin response in infants (*n* = 21) with high risk for celiac disease prospectively followed from birth. Binding of serum antibodies to biotinylated native and deamidated α- and γ-gliadin peptides were measured in PreventCD study participants by bio-layer interferometry on Streptavidin sensors in duplicates. Average values calculated in μg/mL from peptide-specific calibration curves were plotted at the age of 4 months (**a**), 9 months (**b**) and > 2 years (**c**, time of CeD diagnosis). *p* value < 0.05 was taken as significant. **p < 0.01, ***p < 0.001 and ****p < 0.0001 by one-way Anova analysis. Neg.Ctr: irrelevant peptide. Dashes indicate mean values

At the earliest timepoint, we detected anti-gliadin peptide reactivity toward the γ-gliadin peptides, however, these antibodies most probably had maternal origin (all positives had a celiac mother), since infants had not consumed dietary gluten by the time of blood sampling and the own humoral immunity was not fully developed (Fig. 5a).

At the age of 9 months children consumed dietary gluten and began to produce anti-gliadin peptide antibodies that exclusively target the γ-gliadin sequences (Fig. 5b). At the age of 2 years some patients started to produce anti-TG2 antibodies and were subsequently diagnosed with CeD based on duodenal histopathology. At that time the anti-gliadin peptide reactivity strongly increased and p31-43, p57-68 peptide reactivity also appeared, though the majority of antibodies still recognized the γ-gliadin sequences (Fig. 5c). These results clearly indicate that the first responder B-cells are γ-gliadin specific.

### Celiac antibodies affinity-purified by single peptides reveal their cross-reactive nature

Both clinical results and reactivity with overlapping epitopes raised the possibility that the same antibodies might react with several peptides. Therefore, we investigated the possible cross-reactive feature of CeD patients’ gliadin peptide-specific antibodies with affinity-purification from two CeD patient serum samples (only these contained enough high amounts of both α and γ-gliadin reactivities and were available in sufficient volumes) using single gliadin peptide sequences. These polyclonal antibody populations individually purified by different single sequences were then tested for their binding capabilities toward all the used native and deamidated α- and γ-gliadin sequences.

**Fig. 6:**
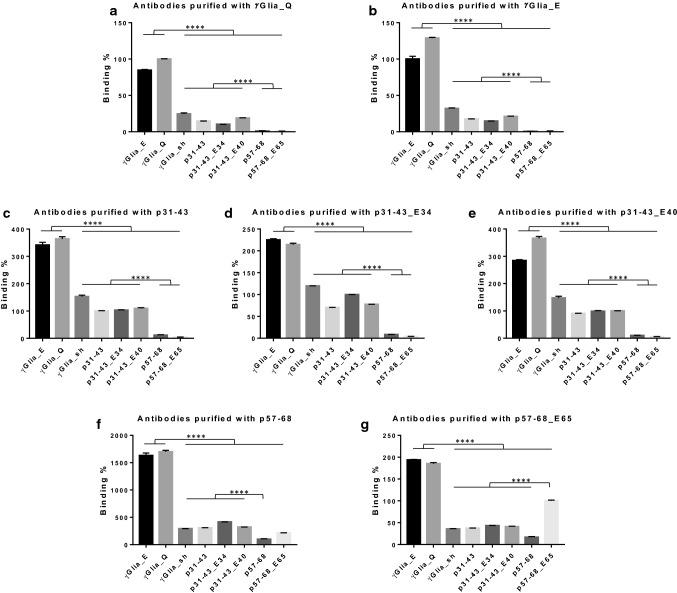
Testing cross-reaction of single gliadin peptide-purified celiac patient-derived antibodies. Antibodies were individually affinity-purified from two celiac disease serum samples with 3 different γ-gliadin (γGlia_Q, γGlia_E, γGlia_sh) and 5 different α-gliadin (p31-43, p31-43_E34, p31-43_E40, p57-68 and p57-68_E65) biotinylated peptides linked to neutravidin beads. Assays were performed by bio-layer interferometry on peptide-coated Streptavidin sensors in duplicates using 150 nM of purified antibodies. Binding rates were calculated by the BLItz Pro 1.2.1.5 software, subsequently averaged and normalized for the binding of antibodies to the peptide wherewith the given antibody reagent was purified and which was set to 100%. *p* value < 0.05 was taken as significant. ****p < 0.0001 by one-way Anova analysis. Bars represent averages with standard errors

Utilizing either γGlia_Q or γGlia_E peptides as the purification antigen, the isolated antibody populations showed overwhelming binding preference for the γ-gliadin peptides, however, displayed considerable binding to the p31-43 peptides (among them highest value with p31-43_E40) as well, while only low-level binding to the p57-68 peptides was observed (Fig. 6a, b). The γGlia_sh peptide was a less potent binding partner for these antibodies, most probably due to its reduced length. Interestingly, antibodies purified with γGlia_E recognized γGlia_E and γGlia_Q equally, confirming the results with the whole serum samples that deamidation did not increase antibody binding.

When we applied any p31-43 peptides as the purification antigen, the extracted antibody populations preferentially recognized the γGlia_Q and γGlia_E sequences compared to α-gliadin sequences clearly indicating their cross-reactive nature (Fig. 6c–e). Furthermore, these antibody populations recognized the p31-43, p31-43_E34, p31-43_E40 and γGlia_sh peptides equally, but reacted poorly with the p57-68 and p57-68_E65 peptides. The difference in their p31-43 and p57-68 recognition suggests that despite the existing cross-reactivity between p31-43 and γ-gliadins, the p31-43 and p57-68 peptides are significantly different antigens for CeD patients and p31-43 peptide is more similar to the preferred γ-gliadin sequences. This similarity might be due to the presence of the PQQ motif in p31-43, while the p57-68 peptide lacks this motif.

Despite the low-level serum reactivity, we were able to isolate sufficient amounts of gliadin-specific antibodies using p57-68 and p57-68_E65 as antigens, respectively. These antibodies also exerted preferential binding to the γGlia_Q and γGlia_E sequences proving their cross-reactivity (Fig. 6f, g). The antibodies purified with p57-68 recognized p31-43 peptides better then p57-68 peptides, indicating that it is a similar antibody population to that we isolated with the p31-43 peptides (Fig. 6f). However, antibodies purified with p57-68_E65 preferred the p57-68_E65 sequence to p31-43 peptides, thus were deamidation-sensitive at this residue (Fig. 6g). Such deamidation sensitivity was also observed with antibodies affinity-purified with the deamidated p31-43_E34 peptides. In both cases, these purified antibodies showed a preference for the deamidated γGlia_E as well, compared to γGlia_Q.

### Effects of α and γ-gliadin peptides in cell culture

**Fig. 7:**
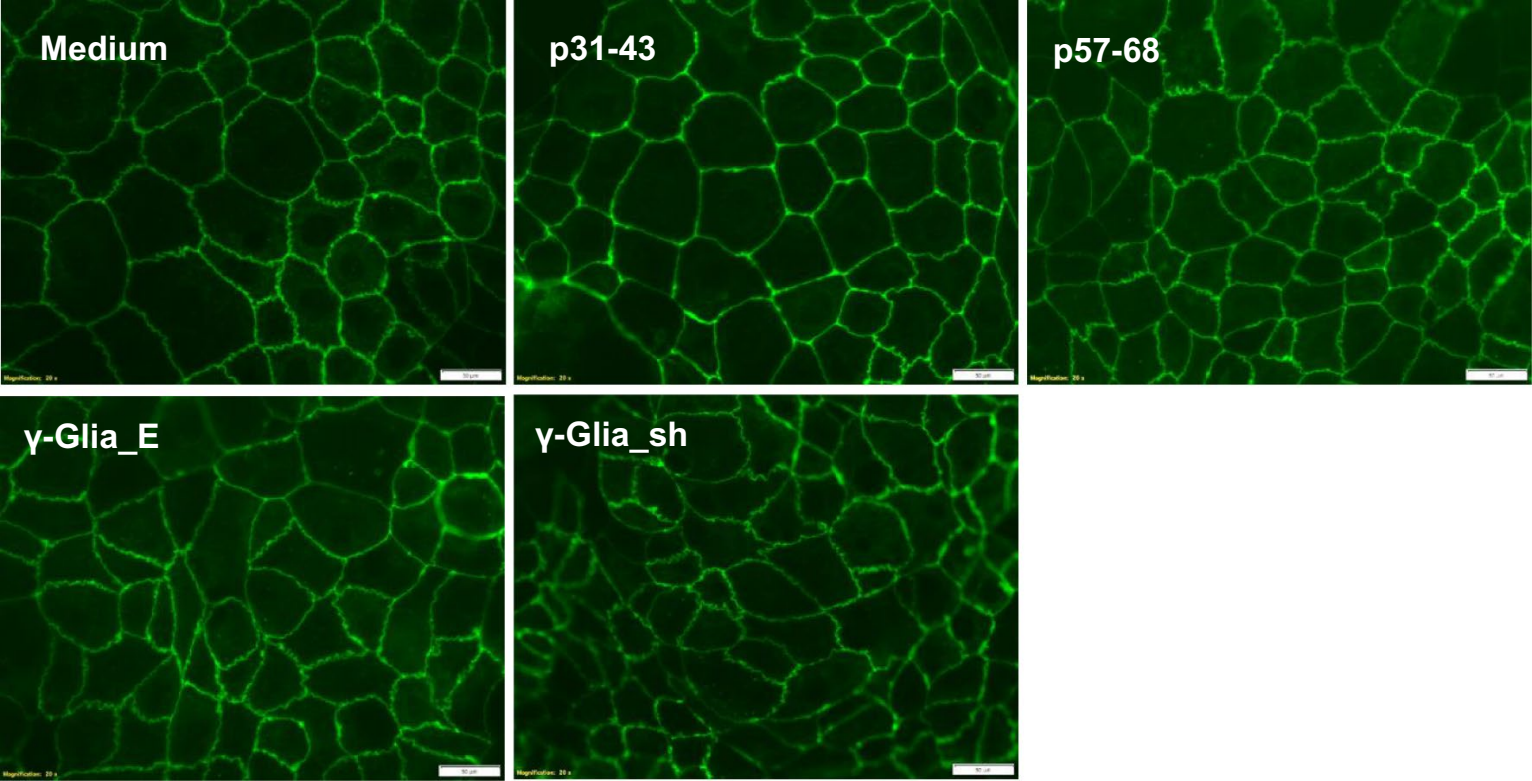
Appearence of tight junctions by anti-ZO1 staining. Caco-2 cells incubated with medium or with different biotinylated α-gliadin (p31-43, p57-68) and γ-gliadin (γGlia_E, γGlia_sh) peptides for 24 h. Representative pictures taken at 250 × magnifications from two independent experiments. Bars represent 50 μm

Since the antibodies against the p31-43 sequences were highly cross-reactive with the γ-Glia peptides dominating the humoral immune response in CeD, we investigated whether the γ-Glia peptides have similar toxic effects on Caco-2 cells as p31-43 peptides. However, neither the p57-68 peptides nor γ-Glia peptides induced loss of tight junction indentations seen by anti-ZO-1 staining in Caco-2 cell culture, a typical toxic effect of p31-43 peptides also obtained in this setting with our biotinylated p31-43 peptides (Fig. 7). The results indicate that these biological effects are not related to the homologous sequences between the p31-43 and γ-Glia peptides.

## Discussion

Based on the literature, α/β and γ-gliadins are equally potent initiators of the T-cell response in CeD (Qiao et al. 2005; Tollefsen et al. 2006; Dørum et al. 2010), however, B-cells seem to prefer reacting to γ-gliadins (Srinivasan et al. 2013; Dørum et al. 2016). Here we have shown by label-free quantitative analysis and by binding studies with single peptide affinity-purified antibodies that, albeit the primary targets for the humoral response are certain γ-gliadin peptides, some of the α-gliadin sequences are antigenic for B-cells, and their antigenicity can be augmented by TG2-mediated deamidation.

The p31-43 and p57-68 peptides are two important α-gliadin fragments in the pathomechanism of CeD. P57-68 is the most potent DQ2-restricted T-cell stimulatory gliadin sequence where TG2-mediated deamidation plays a crucial role for HLA binding (Arentz-Hansen et al. 2000), while 31–43 is a unique α-gliadin fragment that can activate the innate immunity in CeD patients by causing cellular stress (Barone et al. 2014). In accordance with the current knowledge, QXP is the favoured deamidation site for TG2 while QP and QXXP are not good substrates for deamidation (Vader et al. 2002). Most such studies with gliadin substrates originated in early times when only guinea pig TG2 or human recombinant TG2 with glycine at position 224 were available. Other studies relied on the pull-down of stimulatory peptides by gluten-specific T-cells (Dørum et al. 2010). Therefore, we wished to directly determine the resulting peptides from synthetic p31-43 peptides after incubation with recombinant TG2, identical to the naturally occurring human TG2 which has a valine residue at position 224 (Kanchan et al. 2013), since this change influences the enzymatic activity. Mass spectrometry analysis indicated that single or multiple deamidations are indeed generated by TG2 from the p41-43 peptide at Q34 and/or Q40 resulting in QEQPF, PEQPY motifs. In the p57-68 peptide, deamidation at Q65 was the most frequent reaction product, which confirms experimentally the generation of the PELPY motif.

Measuring the binding of serum antibodies to synthetic peptides selected on the basis of these results, antibody amounts directly quantitated by bio-layer interferometry (BLItz) demonstrated remarkable serum reactivity toward the deamidated p31-43 peptide, while p57-68 showed less, however, still detectable signals. Our results show that the highest total serum reactivity is directed toward the γ-gliadin peptides. The γGlia_Q (non deamidated) or γGlia_E (deamidated) sequences are equally good as antigens to measure serum antibody concentrations in high correlation with the clinical DGP antibody assays.

Consequently, no deamidation-dependent binding difference of CeD patients’ gliadin-specific antibodies could be detected for the γ-gliadin sequences, and this result has been confirmed by our studies with affinity-purified antibodies isolated by single peptides. This observation is in line with the earlier findings (Snir et al. 2017) based on IGHV sequencing results in CeD patient-derived gliadin peptide-specific B-cell clones. While clinical assays showed enhanced diagnostic accuracy in earlier studies when deamidated peptides were used as antigens (Schwertz et al. 2004; Rashtak et al. 2008), it must be emphasized that the direct bio-layer interferometry assay ensures better accessibility for antibodies to short peptide sequences compared to enzyme-linked clinical immunoassays. Direct coating of antigens to a solid surface may compromise antibody binding by steric hindrance. In contrast, peptides linked to the SA biosensor via biotin tag and linker sequences in BLItz behave as antigens in the liquid phase. This difference may explain the better binding of antibodies to native peptides in our setting.

Deamidation of p31-43 α-gliadin peptides at Q34 or Q40 residues created sequences highly homologous to shortened γ-gliadin epitopes, but in the case of these very short binding motifs, deamidation-dependent antibody reactivity was indeed found. Although in our experimental setting TG2 was able to deamidate alternatively Q35 in this peptide (Fig S1), the resulting QEPF sequence had been previously experimentally shown to have lower antigenicity for celiac antibodies than the non-deamidated sequence (Schwertz et al. 2004). Therefore we did not use this deamidated peptide in our study.

To our knowledge, this is the first time to take advantage of using different single peptide antigens for antibody purification from CeD patient serum samples and investigate their antigen-binding preferences and cross-reactivity. We found that antibodies purified with any of the antibody-reactive gliadin peptides recognize the QPQQPFP γ-gliadin motifs the most efficiently, and these results suggest that this is a superior immunogen for B-cells. None of the patients had higher reactivity to p31-43 or p57-68 α-gliadins than to γ-gliadins which is also underpinning the dominant role of γ-gliadins in CeD patients’ B-cell immunity. Furthermore, QPQQPFP-containing peptides are involved in the early anti-gliadin peptide immune response in infancy, since the first appearing gliadin peptide-specific antibodies recognize exclusively γ-gliadin peptides.

Based on that, our results suggest that serum reactivity to p31-43 and p57-68 peptides is evolving from cross-reactive γ-gliadin specific antibodies during immune maturation. Gliadins are rich in repetitive sequences and also some commercially used monoclonal mouse anti-gliadin antibody reagents (G12, A1) produced originally against the 33mer α-gliadin fragment do have unusually broad antigen specificity (Morón et al. 2008) and recognize the QPQLPY, QPQQPY, QPQQPF, QPQLPF, QPQLPL, QPELPY motifs as well. Here we report similar cross-reactive nature of CeD patient-derived antibodies arising from originally γ-gliadin reactive B-cells. However, the γ-gliadin peptides in our study did not induce similar toxic effects in cell culture as the p31-43 peptides, thus the biological significance of this cross reactive antibodies or of a possible augmentation of the immune response by α-gliadins is still unclear.

PQQ might be a crucial γ-gliadin derived motif for CeD patients’ gliadin peptide-specific antibodies, as the p31-43 peptide possessing the PQQ motif was a far better antigen in our experiments than p57-68, that lacks PQQ. According to Schwertz et al. (2004), tyrosine is less favourable for CeD patients’ gliadin peptide-specific antibody binding than phenylalanine. The currently existing gliadin-Fab crystal structures (Snir et al. 2017) explain the structural background of this since the phenylalanine fits well into an apolar pocket where the hydroxyl-group of tyrosine would not be favourable. Also in our study, shortening the PFP motif and changing phenylalanine to tyrosine led to a reduction of antibody binding.

α/β-gliadins generally possess only a few PQQs motifs and have tyrosines and phenylalanines in almost equal ratio, while γ-gliadins consist of many PQQ motifs and contain morefold phenylalanines than tyrosines (Table S1), which may explain why the γ-gliadin derived sequences dominate the humoral anti-gliadin immune response.

Eleven of the CeD children participating both in the PreventCD study and in our measurements were included in a study where gliadin-specific T-cell lines and clones were generated from duodenal biopsies and their specificity was extensively tested with large panels of gliadin peptides (Ráki et al. 2017). This gives an unique opportunity to compare target sequences of gliadin-specific T and B cells in the same subject. However, a close correlation was not observed and individual variations were large with cross reactions for several peptides both at the humoral and cellular level (data not shown). From 81 gliadin specific T-cell clones generated from 10 pediatric patients, only 9 were specific for α-gliadins, including the 33-mer highly immunogenic sequence; 22 were specific for γ-gliadin sequences (of them 5 cross-reacting with ω-gliadins), 2 were specific for ω-gliadins (all cross-reactive with γ-gliadins) and for the rest, the exact trigger sequence could not be determined. From the 4 subjects with identified α-gliadin reactive T-cell clones, serum antibodies reactive with p57-68 α-gliadin peptides could be detected in our measurements in only one. On the contrary, the majority of trigger peptides for T-cells reacting to γ and ω-gliadins contained the QPEQPFP sequence or its fragments for which we detected antibodies in all cases.

Although T-cells already in early age showed higher proliferation rates—with only a few exceptions—when stimulated with deamidated peptides compared to non-deamidated peptides (Ráki et al. 2017), in our study serum anti-gliadin antibodies in neither PreventCD participants nor in other CeD patients showed a preferential binding to deamidated γ-gliadin peptides. However, in the case of α-gliadin peptides, deamidated epitope sequences mimicking shortened versions of the γ-gliadin sequences were better recognized by the antibodies than their native counterparts. In other words, shortening of the epitope was better tolerated for maintaining the antibody binding if the sequence contained at least one deamidated residue.

Cross reaction of serum antibodies with several structurally related gliadin peptides also has important clinical implications in diagnostic testing. This feature may substantially enhance the sensitivity of clinical detection, but use of α-gliadins alone as antigens would not be sufficient and, based on our results, it is not likely that (even deamidated) α-gliadin antigens would detect additional antibody populations in young children negative for both anti-TG2 and gliadin antibodies in conventional clinical tests. In contrast, serum antibody quantitation in our study with just one single synthetic peptide containing the deamidated γ-gliadin QPEQPFP motif to which all other gliadin antibodies were cross-reactive, correlated very well with the results obtained for the same samples in six different clinically used commercial DGP IgG assays, although these commercial tests apply multiple and mostly proprietary peptide antigens and current clinical results are expressed only in arbitrary units. All these six assays showed had high predictability for CeD in anti-TG2 positive patients in the multicenter ProCeDE international study (Werkstetter et al. 2017), and despite the low number of negative samples tested here, it can be thus assumed that the bio-layer interferometry would be a similarly useful antibody measurement tool also in clinical practice. Since it measures specific antibody binding directly in μg/mL, BLI would represent important advance for calibration and harmonization of clinical results and for adjusting the measuring range in laboratory practice. In fact, depending on the assay used, 0–39.1% of results exceeded the upper measuring range in those six clinical assays. In such cases, it is difficult to compare follow-up results on diet with the initial values for the purpose to show antibody decrease in response to treatment of CeD.

Another advantage of this label-free method is quantitating both serum IgA and IgG antibodies in one step. In clinical studies, IgG-based tests had higher diagnostic sensitivity while IgA anti-gliadin peptide antibodies were associated with higher accuracy (Rashtak et al. 2008) and with very high values in young children with CeD (Vriezinga et al. 2014). IgG anti-gliadin peptide antibody values may be, however, low in many untreated CeD cases, especially in older children and adults, and the testing is less specific than anti-TG2 assays. This study did not investigate the diagnostic accuracy of the antibody populations against different gliadin peptides. However, the observed cross-reaction to structurally similar but non-related peptide motifs in gluten-free cereals (data not shown) may explain that non-specific positivity for gliadin antibodies is not rare in clinical practice.

In conclusion, we observed that while γ-gliadins are the main targets of celiac antibodies, the humoral immune response also can involve the investigated α-gliadin peptides by cross-reaction. This especially happened if they were used in specifically deamidated forms similar to those that could be experimentally generated by human TG2.

## Supplementary Information

Below is the link to the electronic supplementary material.

Supplementary file1 (DOCX 333 KB)

## Data Availability

The datasets generated and/or analysed during the current study are available from the corresponding author on reasonable request.

## Abbreviations

BLI: Biolayer interferometry
CeD: Celiac disease
DGP: Deamidated gliadin peptide
FBS: Fetal bovine serum
HLA: Human leukocyte antigen
LC–MS/MS: Liquid chromatography with tandem mass spectrometry
PBS: Phosphate buffered saline
PBST: Phosphate buffered saline + 0.1% Tween 20
SA: Streptavidin-coated biosensor
TG2: Type-2 (tissue, cellular) transglutaminase

## Code availability

PRISM 7.00 software (RRID: SCR_005375). Scaffold proteome software (RRID: SCR_014345). BLItz Pro 1.2.1.5 software.
