# Supplementary material for: Gamma-gliadin specific celiac disease antibodies recognize p31-43 and p57-68 alpha gliadin peptides in deamidation related manner as a result of cross-reaction
Source: Amino Acids. 2021 May 31;53(7):1051–63. doi: 10.1007/s00726-021-03006-7 (PMC8241804; doi:10.1007/s00726-021-03006-7)

**Supplementary material**

Gamma-gliadin specific celiac disease antibodies recognize p31-43 and p57-68 alpha gliadin peptides in deamidation related manner as a result of cross-reaction

Authors: Ádám Diós, Rita Elek, Ildikó Szabó, Szilvia Horváth, Judit Gyimesi, Róbert Király, Katharina Werkstetter, Sibylle Koletzko, László Fésüs, Ilma R. Korponay-Szabó

**Supplementary figure 1.** MS/MS analysis of transglutaminase 2-mediated deamidation of α-gliadin peptides

*Peptide 31-43*

S1A. 50 pmol of human recombinant TG2 enzyme were incubated with 150 fold molar excess of the p31-43 peptide for 2h at 37⁰C. The resultant deamidation products were detected with LC-MS/MS analysis. +1 and +2 indicate b or y-fragments on the spectrum containing one or two deamidations respectively. Deamidation at position Q40 was abundant in the peptide fragments while deamidation of Q35 position was very rare based on Scaffold analysis.

*Peptide 57-68*

S1B. 50 pmol human recombinant TG2 enzyme was incubated with 150 fold molar excess of the p57-68 peptide for 2h at 37⁰C. The resultant deamidation products were detected with LC-MS/MS analysis. +1 and +2 indicate b or y-fragments on the spectrum consisting one or two deamidations respectively. Deamidation at Q65 position was abundant in the peptide fragments, while Q59 deamidation was very rare.

**Supplementary Figure 2.** Correlation of bound antibody values in μg/mL to the QPEQPFP (yGlia_E) deamidated γ-gliadin epitope measured by bio-layer interferometry with results of six commercial clinical anti-DGP IgG antibody measuring assays using serum samples from 69 untreated celiac patients.

D1-D6: commercial IgG DGP antibody assays included in the ProCeDE study (Werkstetter et al. 2017). Assays D1,D3,D4,D5 and D6 had calibration curve-based antibody concentration calculation in arbitrary units (AU), whereas in D2 assay, results are calculated as multiples of optical density of a cut-off sample, so numeric results are logaritmic derivatives of antibody concentration. Correlation analysis was performed by Spearman’s rank correlation test. Samples with values exceeding the highest calibrator and thus being outside the measuring range of the assay were excluded (0 in D1-D3, 27 in D4, 17 in D5 and 18 in D6).

**Supplement Table 1.** Difference in amino acid composition of α- and γ-gliadins

Columns show the average number of PQQ motifs and the F : Y ratio referring to 100 aminoacids in α- and γ-gliadins. Data are based on UniProt KB reviewed protein sequences from *Triticum aestivum*. Representative sequence accession numbers: P02863 for α/β-gliadins; P08453 for γ-gliadins. Positions of peptides utilized in this study are marked by underlining.


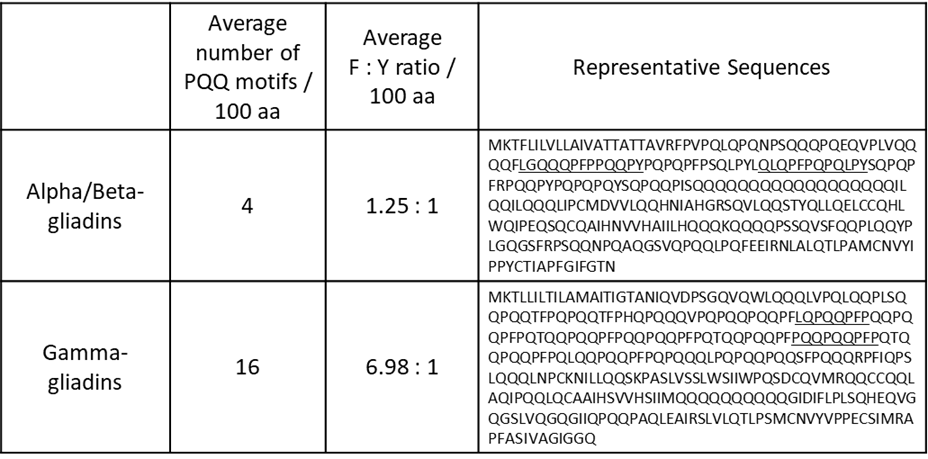

Supplement: Supplementary file 1 — Supplementary file1 (DOCX 333 KB) [file 726_2021_3006_MOESM1_ESM.docx]
